# *CmLOX10* positively regulates drought tolerance through jasmonic acid -mediated stomatal closure in oriental melon (*Cucumis melo* var.* makuwa* Makino)

**DOI:** 10.1038/s41598-020-74550-7

**Published:** 2020-10-15

**Authors:** Qiaojuan Xing, Jingjing Liao, Songxiao Cao, Meng Li, Tinghui Lv, Hongyan Qi

**Affiliations:** College of Horticulture, Shenyang Agricultural University/Key Laboratory of Protected Horticulture, Ministry of Education/Northern National & Local Joint Engineering Research Center of Horticultural Facilities Design and Application Technology (Liaoning), Shenyang, 110866 China

**Keywords:** Physiology, Plant sciences

## Abstract

Drought stress severely impairs plant growth and production. Lipoxygenase (LOX), a master regulator for lipid peroxidation, is critical for direct or indirect response to abiotic stresses. Here, we found that drought stress induced the transcription of *CmLOX10* in leaves of oriental melon seedlings. Reverse genetic approaches and physiological analyses revealed that silencing *CmLOX10* increased drought susceptibility and stomatal aperture in oriental melon seedlings, and that ectopic overexpression of *CmLOX10* in *Arabidopsis* enhanced drought tolerance and decreased the stomatal aperture. Moreover, the transcription of jasmonic acid (JA)-related genes and JA accumulation were significantly induced in *CmLOX10-*overexpressed *Arabidopsis*, which were reversely suppressed in *CmLOX10-*silenced seedlings during the stage of drought stress. Foliar application of JA further verified that JA enhanced drought tolerance and induced stomatal closure in leaves of melon seedlings. In addition, the feedback regulation of *CmLOX10* was induced by JA signaling, and the expression level of CmMYC2 was increased by JA and drought treatment. Yeast one-hybrid analysis showed that CmMYC2 directly bound to the promoter of *CmLOX10*. In summary, we identified the important roles of C*mLOX10* in the regulation of drought tolerance in oriental melon seedlings through JA- mediated stomatal closure and JA signaling-mediated feedback through CmMYC2.

## Introduction

Drought stress is a major environmental stress that severely impairs plant growth and development, even leading to a serious loss in crops yield^1^. Extensive research has shown that the defense mechanism in drought stress is related to the expression of stress-related genes, the accumulation of metabolites, the synthesis of osmoprotectants and antioxidants, the maintenance of root growth and water uptake, and the modification of transpiration water loss^2^. In addition, a large number of studies indicated that phytohormones are important in the drought stress response.

Abscisic acid (ABA), a sesquiterpene derived from C40 oxygenated carotenoids through several synthesis steps^3^, plays critical roles in drought tolerance in plants^4^. ABA is involved in physiological processes, such as stomatal closure, osmolyte accumulation, and the synthesis of stress-related proteins ^5^. In recent years, numerous studies suggested JA plays an important role in response to abiotic stresses. Exogenous application of MeJA promoted plant resistance to abiotic stress, namely salt stress ^6^, chilling ^7^, drought stress ^8^ and so on. Drought stress induced the accumulation of JA ^9^ in plants, then JA enhanced drought stress mainly through regulating stomatal closure and transpirational water loss ^10–13^. Treatment with 12-OPDA, a kind of JA precursor, promoted stomatal closure and OPDA levels in leaf of *A. thaliana*, thereby reducing stomatal aperture and exhibiting higher tolerance on drought stress ^14^.

Lipoxygenases (LOXs), an essential enzyme for JA synthesis, can catalyze the oxygenization of polyunsaturated fatty acids containing a cis, cis-1, 4- pentadiene ^15^, and are classified into 9-LOXs and 13-LOXs in plants according to the specific oxidation sites in linoleic acid ^16^. LOXs play key roles in all kinds of plant physiology process including growth and development ^17,18^, senescence ^19^, and especially responses to biotic and abiotic stresses ^20^. Pepper *CaLOX1* and persimmon *DkLOX3*, belonging to 9-LOX genes, responded to drought and high salinity stress by modulating the expression of ABA- and stress-responsive genes, lipid peroxidation and ROS accumulation in *Arabidopsis* ^21,22^. Numerous studies suggested that 13-LOX genes were mainly involved in the response of plants to biotic and abiotic stresses by regulating JA synthesis. Overexpression of *TomloxD* in transgenic tomatoes resulted in a significant increase of lipoxygenase activity and endogenous JA content, and boosted the tolerant of tomatoes plants to *Cladosporium fulvum* and high-temperature stress ^23^. *Arabidopsis LOX2* was found to contribute to the majority of JA synthesis upon wounding and osmotic stress. *Arabidopsis lox3* mutant displayed salt sensitivity in different developmental stages, and this phenotype could be rescued by MeJA ^6^. Little is known about the physiological and molecular mechanisms of LOXs response to abiotic stresses in oriental melon.

Previous studies have shown that JA biosynthesis is also regulated by JA signaling mediated-feedback. JA biosynthetic genes, *LOX2* and AOS, were induced by MeJA in *Arabidopsis* ^24^. Several evidences have showed that *AtMYC2* functioned in JA accumulation induced by through directly binding to the promoters of JA biosynthesis and catabolism genes ^25^. The expression of *TomLoxD*, *OPR3*, *AOC*, and *AOS* decreased in the *MYC2*-silenced tomato plants ^26^. In addition, *MYC2* was found to bind to *LOX2/3/4*, *AOS*, and *OPR3*^27,28^ according to the ChIP-qPCR and ChIP-seq analyses.

However, the mechanism of CmLOXs genes in oriental melon, responding to drought stress, except for *CmLOX13* enhancing drought tolerance via regulating ABA accumulation and stomatal closure in *Arabidopsis*, has been unclear. In our previous study, a total of 18 CmLOXs genes were identified from the oriental melon 'YMR' genome ^29^ In this study, we found many LOXs genes are dramatically upregulated under drought stress. Drought stress induced the transcription of *CmLOX10* in leaves of oriental melon seedlings. Reverse genetic approaches and physiological analyses revealed that *CmLOX10* positively affected the response of oriental melon on drought stress and also regulates a JA-mediated pathway in oriental melon seedlings.

## Results

### Expression pattern of CmLOXs in response to drought stress

**Figure 1:**
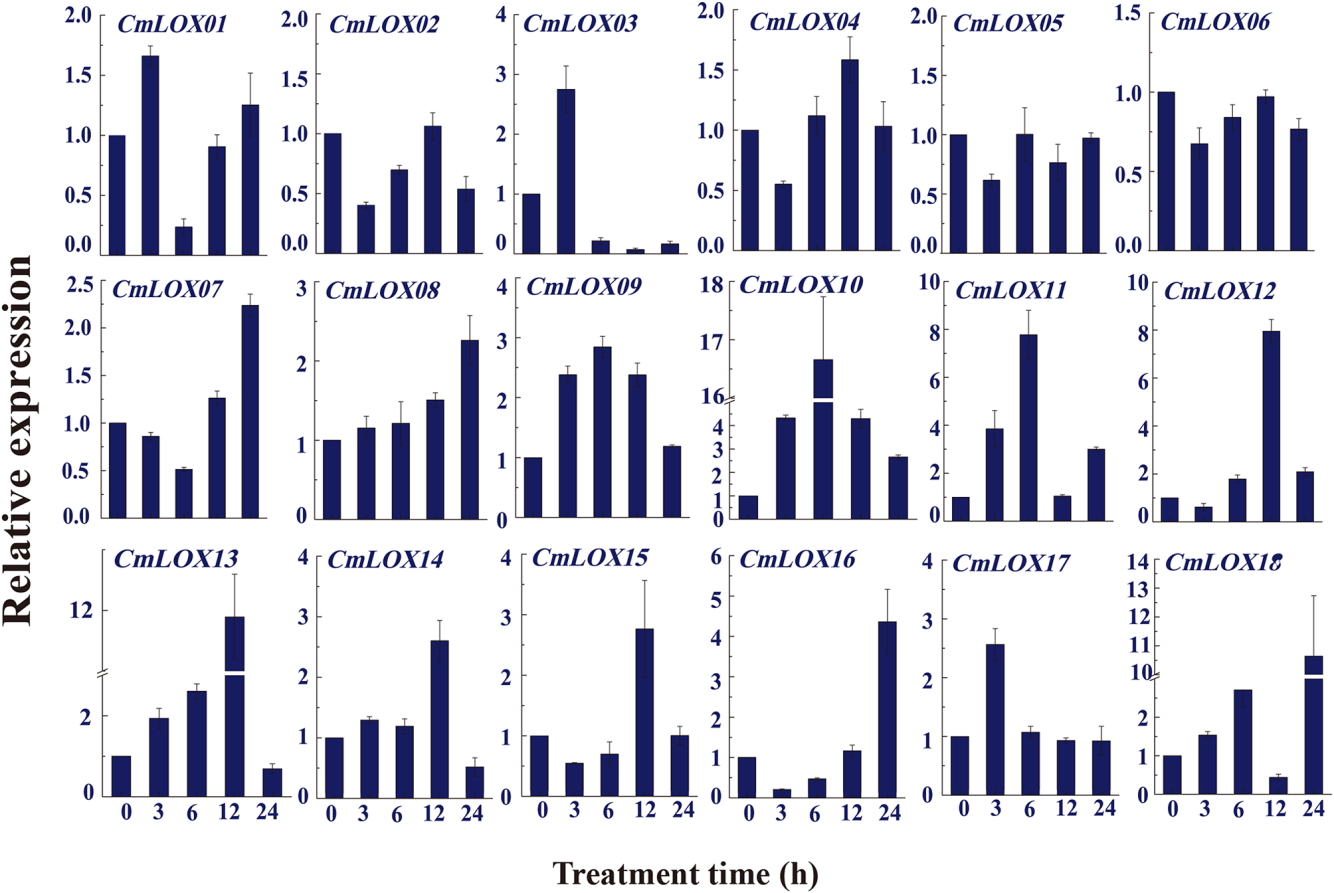
Expression of eighteen CmLOXs genes at various time points under 8% PEG6000 treatment. The expression of 18S rRNA was used as internal control. The test was repeated three times, and one biological repeat including four plants.

Our previous study identified 18 *CmLOXs*, named *CmLOX01-18*, and five of them were differently induced by abiotic and signal molecules stresses, such as wounding, high temperature, MeJA, ABA and H_2_O_2_^29,30^. In this study, we were interested in characterizing the role of CmLOXs in response to drought stress. qRT–PCR analysis has shown that the transcription of *CmLOX10, CmLOX11, CmLOX12, CmLOX13,* and *CmLOX18* in oriental melon leaves were strongly induced by 8% PEG6000 treatment, while the expressions of *CmLOX02, CmLOX05* and *CmLOX06* were almost unchanged, and the other CmLOXs were slightly up-regulated by 8% PEG6000 treatment (Fig. 1). In this study, we determined to further explore the function of the *CmLOX10* in response to drought stress.

### Silenced of *CmLOX10* reduced drought tolerance in oriental melon

**Figure 2:**
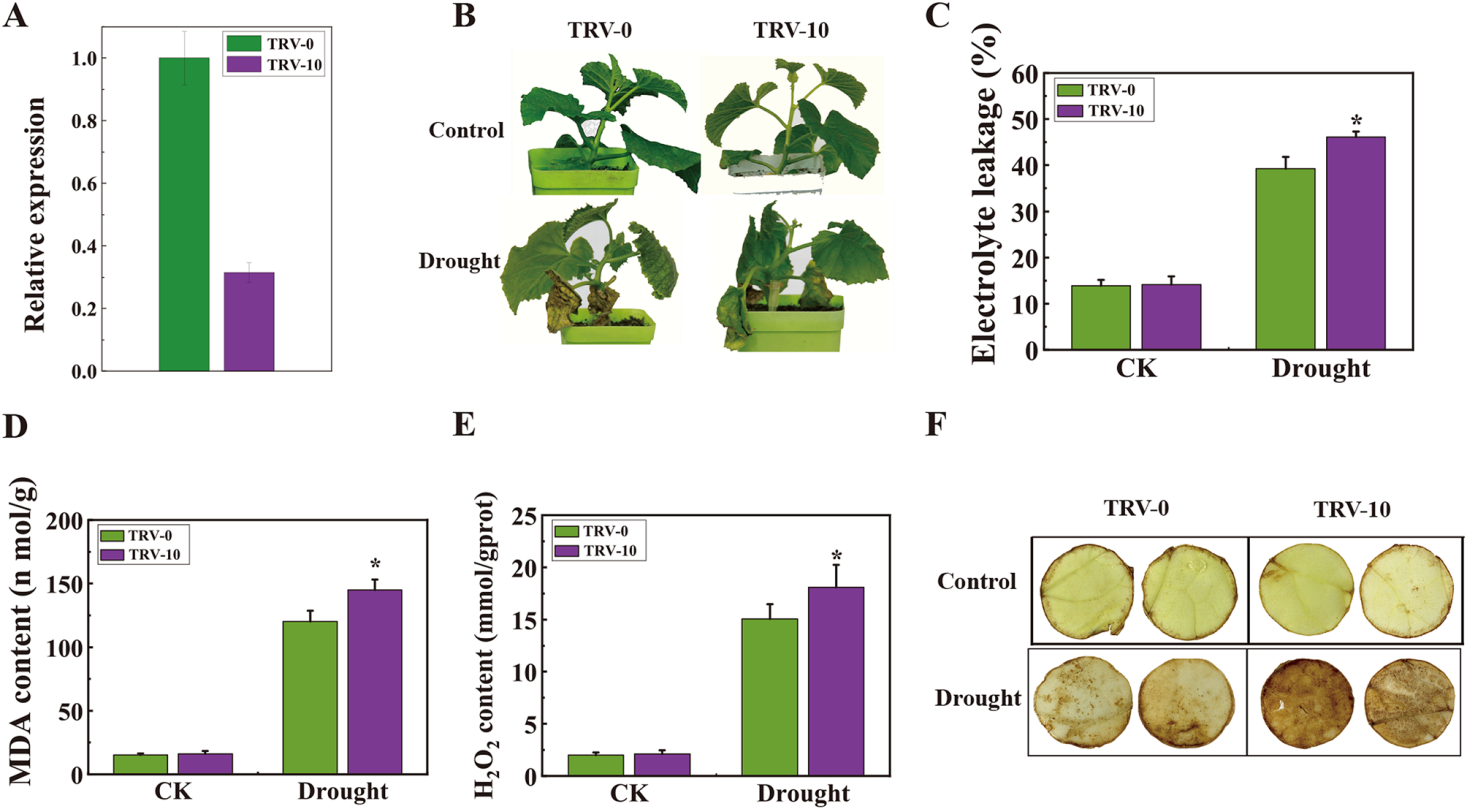
Silencing *CmLOX10* in oriental melon decreased drought tolerance. (**A**) qRT-PCR analysis of *CmLOX10* gene expression in *CmLOX10*-silenced and control oriental melon seedlings. The expression of 18sRNA was used as an internal control. (**B**) Representative phenotypes of control (TRV-0) and *CmLOX10*-silenced (TRV-10) plants at the four-leaf stage after 10 d of drought treatment. Electrolyte leakage (**C**) and MDA contents (**D**), representative photographs showing DAB staining (**E**) and H_2_O_2_ contents (**F**) in TRV-0 and TRV-10 plants under control and drought conditions. Error bars show the SD for n = 3. Asterisks indicate significant differences between the TRV-0 and TRV-10 constructs (Student’s t test, *P < 0.05).

To investigate the loss-of-function phenotype of *CmLOX10* in drought response, we utilized a *tobacco rattlev virus* (TRV)-induced gene silencing (VIGS) technique to knock down the *CmLOX10* gene in oriental melon plants. During four weeks after VIGS, qRT-PCR analysis results verified that *CmLOX10* expression level was significantly decreased in the pTRV-*CmLOX10* seedlings (Fig. 2A). TRV-10 seedlings displayed no obvious differences in the growth performance and physiology of TRV-0 seedlings under normal growth conditions (Fig. 2B-F). However, after 10 days of drought stress by withholding water, *CmLOX10*-silenced (TRV-10) seedlings exhibited wilting to a greater extent than that of empty vector control (TRV-0) (Fig. 2B). Previous studies have shown that malondialdehyde (MDA) production can be induced by drought stress, and there is a negative correlation between MDA content and drought resistance ^31^. Electrolyte leakage (EL), an important indicator of plant cell permeability, reflects the degree of membrane damage. Besides, under stresses, plants inevitably produced reactive oxygen (ROS), whose accumulation in cells caused oxidative damage to membranes (lipid peroxidation), proteins, RNA and DNA molecules ^32,33^. In this study, drought stress led to drastic accumulation of EL and MDA contents in TRV-10 seedlings, which was higher than that in TRV-0 seedlings (Fig. 2C-D). The results of DAB staining showed few brown spots both on the TRV-0 and TRV-10 leaves under normal condition, while the brown spots on TRV-10 leaves were significantly more than those on the TRV-0 seedlings after drought treatment (Fig. 2E). The H_2_O_2_ contents were consistent with the staining results (Fig. 2F). Taken together, the *CmLOX10* gene may play a positive role in regulating drought tolerance in oriental melon.

### Ectopic expression of *CmLOX10* enhanced Arabidopsis drought tolerance

**Figure 3:**
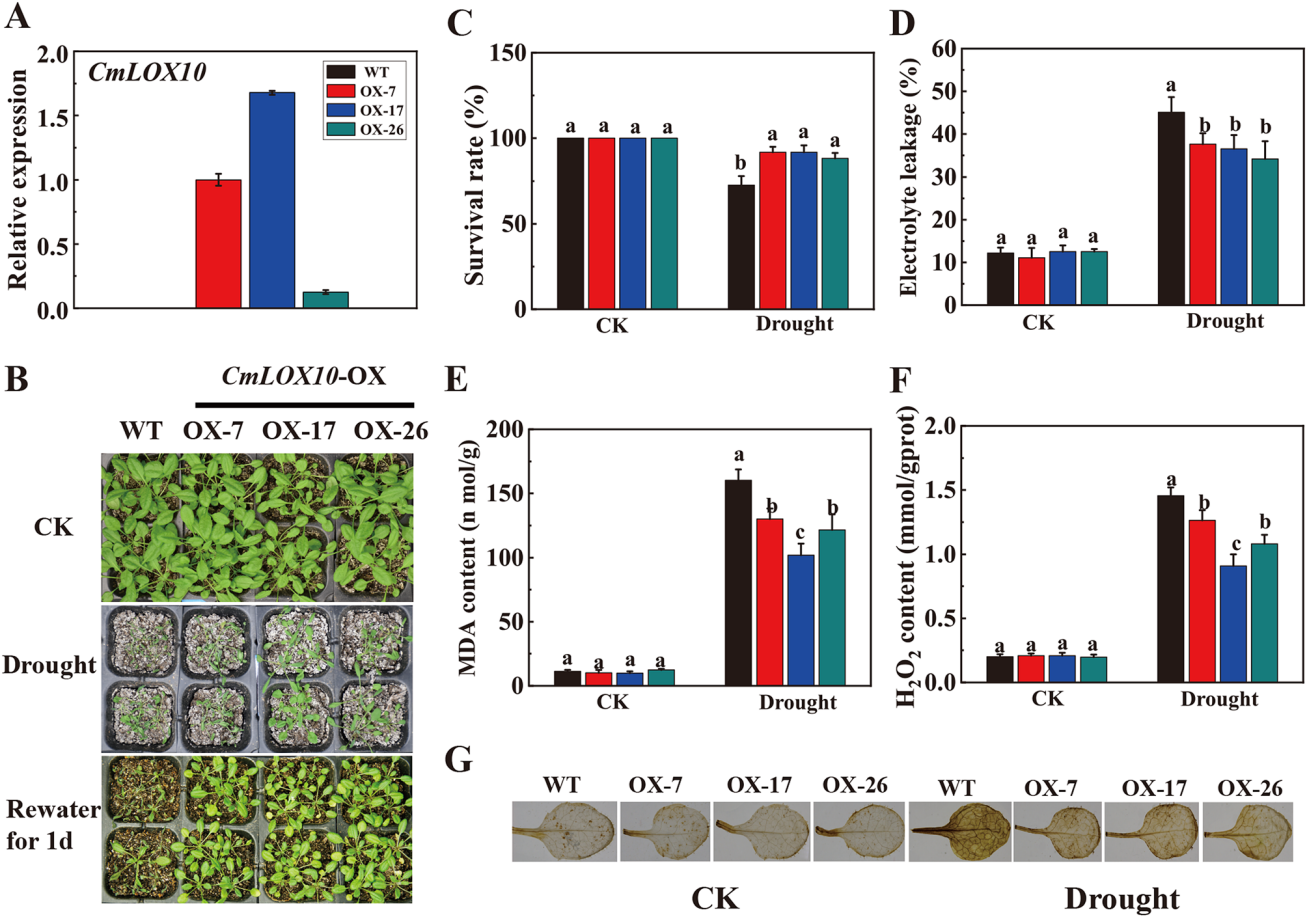
Ectopic expression of *CmLOX10* in *Arabidopsis* enhanced drought tolerance. (**A**) qRT-PCR analysis of *CmLOX10* gene expression in *CmLOX10*-OX and WT Arabidopsis plants. The expression of *Actin7* was used as an internal control. (**B**) Representative phenotypes of WT and transgenic plants under normal and drought conditions. Then watering for one day to allow the plants to recover. (**C**) Survival rates were calculated for the seedlings after 1d of re-watering following drought treatment. Electrolyte leakage (**D**) and MDA level (**E**), representative photographs showing DAB staining (**F**) and H_2_O_2_ level (**G**) in WT and transgenic plants under control and drought conditions. WT, wild type; OX-7, OX-17 and OX-26, individual overexpression line. Error bars show the SD for n = 3. Different letters indicate significant differences at P < 0.05 (Duncan’s multiple range test) of levels for the same index in WT, OX-7, OX-17 and OX-26 plants.

To investigate the gain-of-function phenotype of the *CmLOX10* in drought response, three *CmLOX10*-OX transgenic *Arabidopsis* lines, OX-7, OX-17 and OX-26 were generated (Fig. 3A). There were no differences between *CmLOX10*-OX plants and wild-type plants under normal growth conditions (Fig. 3B–G). 24-day-old of WT and transgenic plants were withheld watering for 10 d, obvious differences were found in growth and physiology between WT and *CmLOX10*-OX plants. Comparing to transgenic plants, more WT plants exhibited wilting symptoms (Fig. 3B). After re-watering for 1 day, survival rates of three transgenic lines were above 88%, being 20% higher than that of the wild type (Fig. 3C). Furthermore, physiological results showed that EL, MDA and the H_2_O_2_ contents in transgenic plants were significantly lower than that in WT plants without watering for 10 days (Fig. 3D–G), which supported the higher survival rates of transgenic plants under drought stress. These results confirmed that ectopic expression of *CmLOX10* enhanced *Arabidopsis* drought tolerance.

### *CmLOX10* positively regulates stomatal aperture

**Figure 4:**
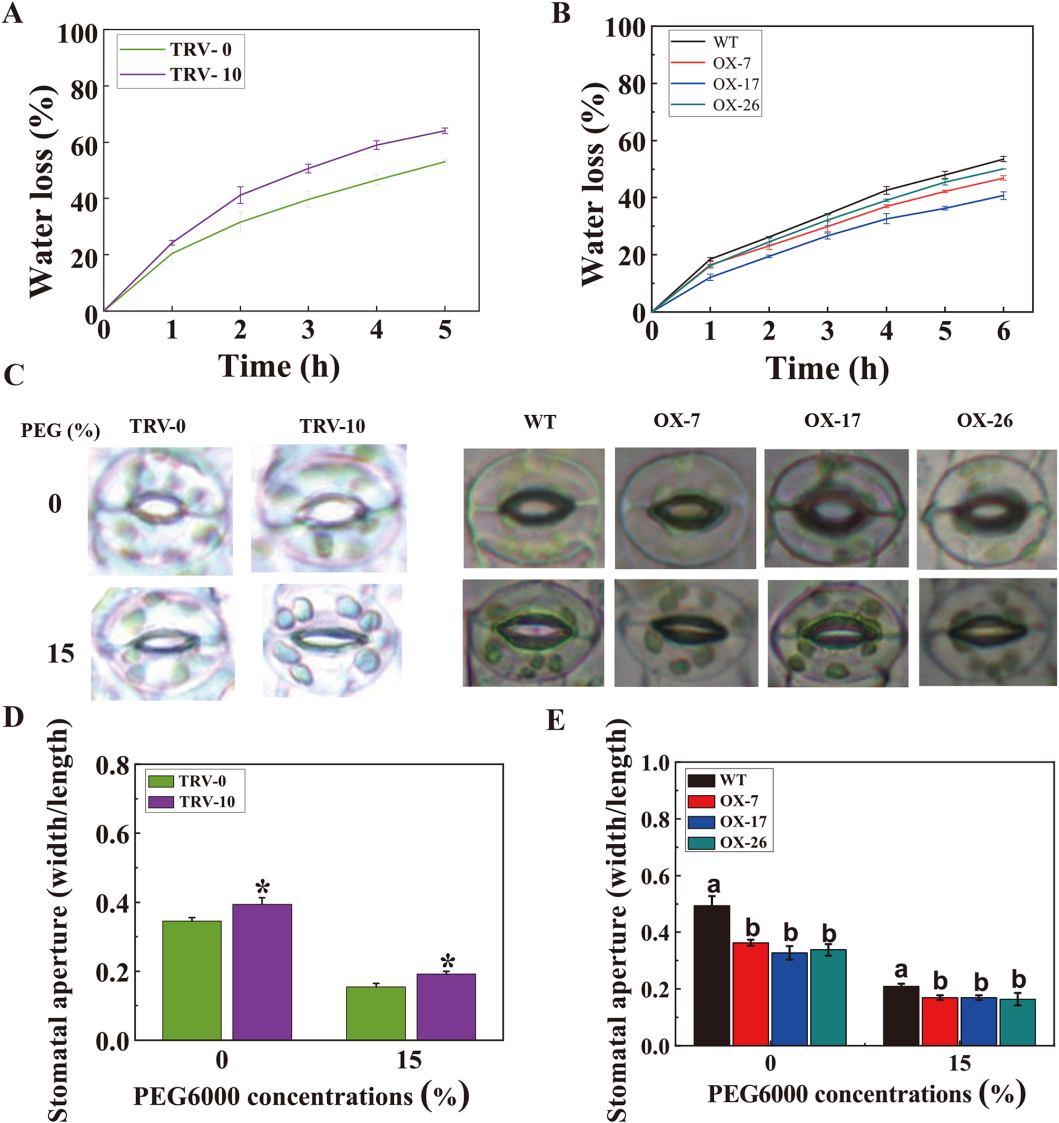
*CmLOX10* contributes to reducing stomatal aperture. (**A**) Water loss from the leaves of TRV-0 and TRV-10 plants at various times (one-hour interval) after leaf detachment. (**B**) Water loss from the leaves of WT and *CmLOX10*-OX plants at various times (one-hour interval) after leaf detachment. (**C**) Stomatal phenotype of *CmLOX10*-silenced and *CmLOX10*-overexpressed plants under different concentrations of PEG (0 and 15%), TRV-0 and WT plants as the control. (**D**) Stomatal size of TRV-0 and TRV-10 plants under different concentrations of PEG (0 and 15%). (**E**) Stomatal size of WT and *CmLOX10*-OX plants under different concentrations of PEG (0 and 15%). Error bars show the SD for n = 3. Asterisks indicate significant differences between the TRV-0 and TRV-10 constructs (Student’s t test, *P < 0.05). Different letters indicate significant differences at P < 0.05 (Duncan’s multiple range test) of levels for the same index in WT, OX-7, OX-17 and OX-26 plants.

Root growth and water evaporation are the two major factors that are involved in the drought tolerance of plant. The root length had no differences between *CmLOX10*-OX and WT plants (Supplementary Fig. S1A), or between TRV-0 and TRV -10 seedlings (Supplementary Fig. S1B). However, Water loss rate, an important indicator of drought stress was higher in TRV-10 leaves than that in TRV-0 leaves (Fig. 4A), and the leaves fresh weight losses of *CmLOX10*-OX transgenic plants were lower than those of WT (Fig. 4B). Water is lost from plant leaves through stomata, reminding us to measure the stomatal size. Larger stomatal aperture was observed in leaves of TRV-10 seedlings, comparing to TRV-0 seedlings, under normal and drought conditions (Fig. 4C, D). Meanwhile, the stomatal aperture in leaves of *CmLOX10*-OX seedlings were smaller than that of WT plants under normal and drought conditions (Fig. 4C, E). Therefore, these results implied that *CmLOX10* could enhance the resistance of plants on drought stress, which may be related to reducing transpirational water loss through decreasing stomatal aperture.

### *CmLOX10* enhanced drought tolerance by regulating endogenous JA synthesis

**Figure 5:**
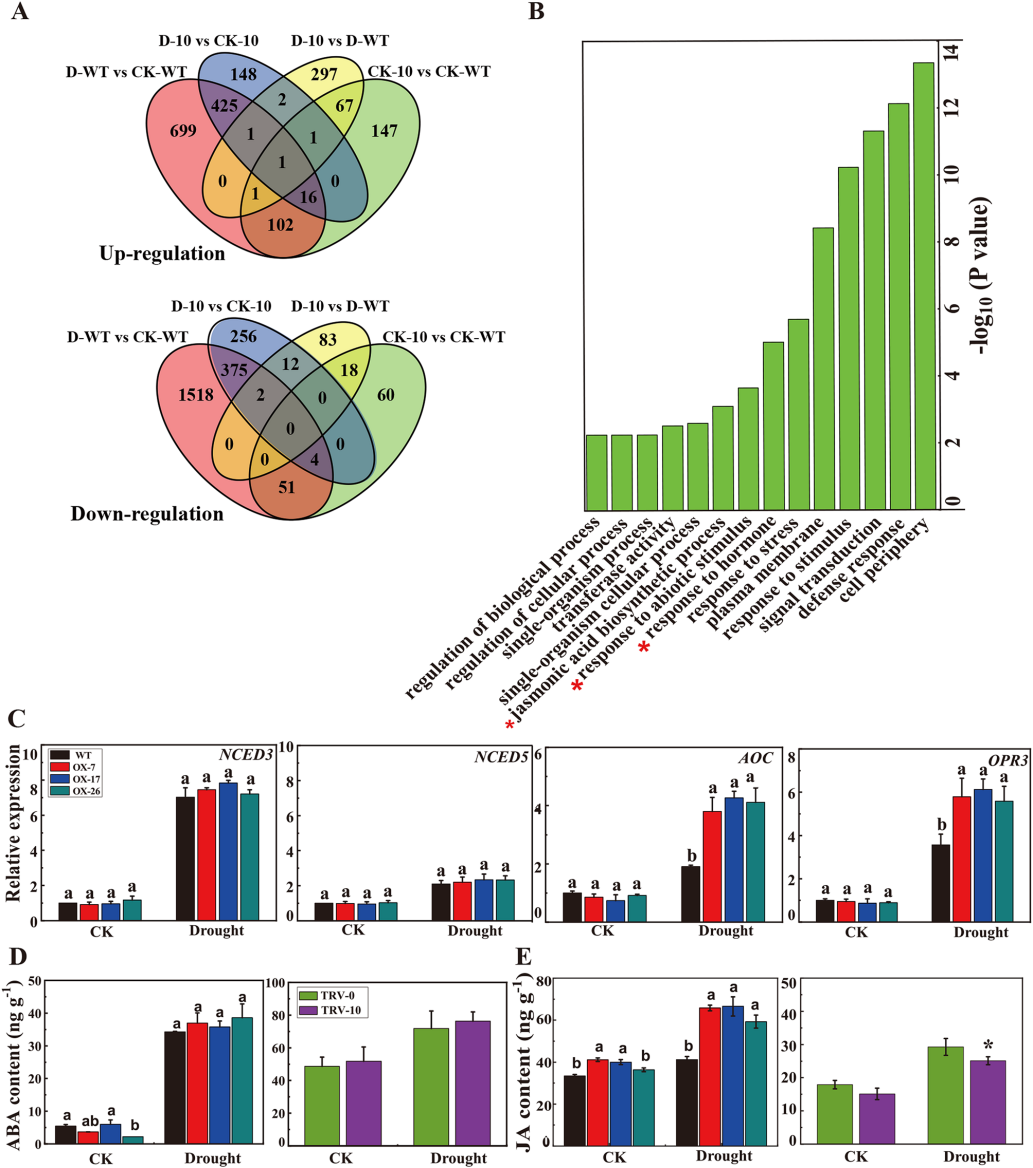
*CmLOX10* increased JA synthesis under drought conditions. (**A**) Venn diagrams of differentially expressed genes by RNA-seq analysis. (**B**) GO analysis of Up-regulation genes in *CmLOX10*-OX plants comparing to WT in biological process under drought condition. (**C**) Expression of key genes in JA and ABA synthesis in WT and *CmLOX10*-OX lines under normal and drought conditions analyzed by qRT-PCR. (**D**) ABA contents in *Arabidopsis* (WT and *CmLOX10*-OX lines) and oriental melon (TRV-0 and TRV-10) under normal and drought conditions. (**E**) JA contents in *Arabidopsis* (WT and *CmLOX10*-OX lines) and oriental melon (TRV-0 and TRV-10) under normal and drought conditions. AOC, allene oxide cyclase; OPR, 12-OPDA reductase; NCED, 9-cis-epoxycarotenoid dioxygenase. Error bars show the SD for n = 3. Different letters indicate significant differences at P < 0.05 (Duncan’s multiple range test) of levels for the same index in WT, OX-7, OX-17 and OX-26 plants.

To explore the role of *CmLOX10* in response to drought stress, four samples, including *CmLOX10*-OX and WT *Arabidopsis* plants under normal and drought conditions with three biological replicates, were used for RNA-seq analysis. Gene ontology (GO) analysis discovered that the DEGs (Fig. 5A) were significantly enriched in biological process including “response to hormone,” “response to abiotic stimulus,” and “jasmonic acid biosynthetic process” between *CmLOX10*-OX and wild-type plants under drought condition (Fig. 5B). Moreover, the pathways analysis displayed many genes affected by *CmLOX10* were involved in diverse pathways. Only hormone metabolism pathway was consistently greater by at least fourfold normalized frequency values in all four comparisons (Table 1). As we all know, ABA and JA (a metabolite of LOXs) are two important phytohormones involved in regulating stomatal closure in response to drought stress in plants ^34^. Interestingly, the expression of the key genes, *NCED3* and *NCED5*, involved in the synthesis of ABA, had no significant difference in transgenic plants from that in WT leaves, while JA biosynthetic-related genes, *AOC3* and *OPR3*, were upregulated in the *CmLOX10*-OX lines comparing to WT during drought stress according to the transcriptional data (Fig. 5C, Supplementary Table S2), which prompts us to determine the endogenous ABA and JA levels in transgenic *Arabidopsis* and oriental melon under normal and drought conditions*.* As shown in Fig. 5D, there was no significant difference of ABA content between *CmLOX10*-OX or TRV-0 plants with control plants. JA contents in *CmLOX10*-OX plants were significantly higher than those of control plants, whereas, JA contents in TRV-10 seedlings were obviously lower than those of control seedlings after drought treatment, respectively (Fig. 5E). These results indicate that *CmLOX10* may improve drought tolerance by increasing the synthesis of endogenous JA.

**Table 1:**
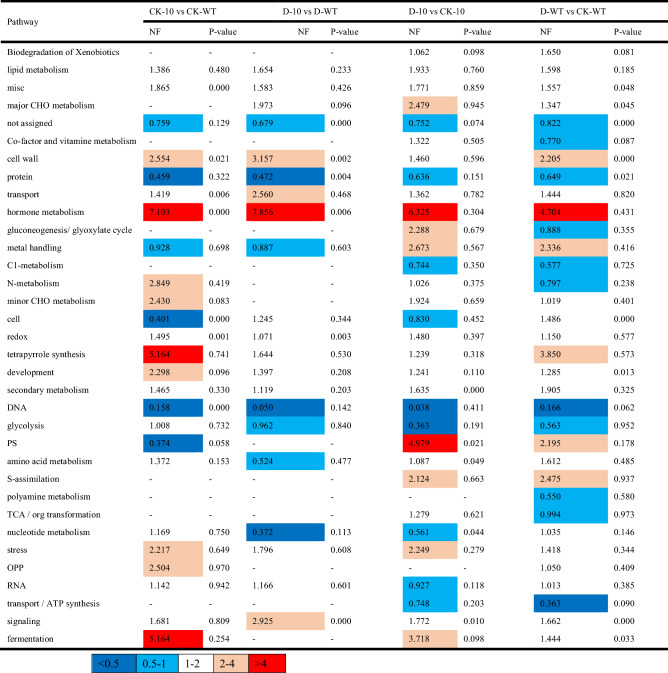
MapMAN pathway enrichment analysis of four types of comparisons from WT and transgene RNA-seq under normal and drought stress conditions.

In order to explore whether *CmLOX10* affects ABA signaling, we first used qRT–PCR analysis to examine the expression pattern of the *CmLOX10* gene after ABA treatment. The results showed that the expression of *CmLOX10* was not induced by exogenous ABA treatment (Supplementary Fig. S2A). Then, *CmLOX10*-OX seeds were germinated on 1/2 Murashige and Skoog (MS) medium with different concentrations of ABA. The germination rate, green cotyledon and root length of *CmLOX10*-OX plants had no significant difference compared with WT plants (Supplementary Fig. S2B, C). In addition, stomatal aperture was measured after ABA treatment, which plays an important role in stomatal closure. However, there was no significant difference in the relative stomatal closure between *CmLOX10*-OX plants and WT plants(Supplementary Fig. S2D), suggesting that the low transpiration rate of *CmLOX10*-OX plants was not caused by ABA- mediated stomatal closure.

**Figure 6:**
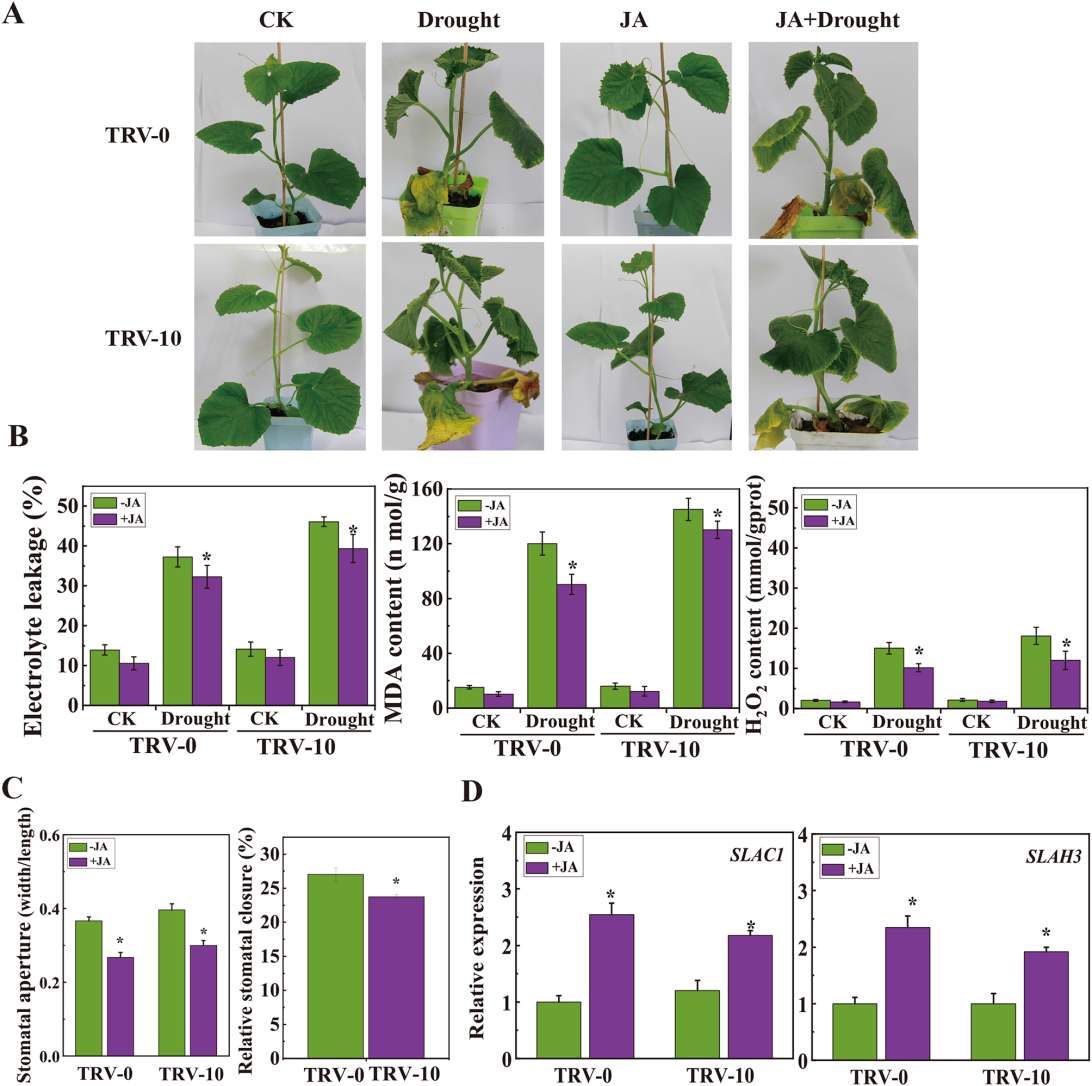
Exogenous addition of JA can alleviate the damage of drought to oriental melon. The “four leaves stage” oriental melon seedlings were treated with 50 μmol L^−1^ JA, 15%PEG6000, 50 μmol L^−1^ JA + 15%PEG6000 for three days, then representative phenotypes (**A**), and Electrolyte leakage, MDA content and H_2_O_2_ level (**B**) were detected. (**C**) Stomatal aperture of TRV-0 and TRV-10 plants under different concentrations of JA (0 and 10 μM). (**D**) Expression of marker genes in regulating stomatal closure in TRV-0 and TRV-10 plants under different concentrations of JA (0 and 50 μM). Error bars indicate SD for three measurements. Asterisks indicate significant differences between the control (TRV-0) and *CmLOX10*-silenced (TRV-10) constructs (Student’s t test, *P < 0.05).

To investigate the relationship between drought stress and JA level, exogenous JA was applied to oriental melon seedlings before drought treatment. Under normal condition, TRV-0 and TRV-10 seedlings grew very well, and the EL, MDA and H_2_O_2_ levels of TRV-10 were similar to those of TRV-0 seedlings. Exposing to drought condition, both TRV-0 and TRV-10 seedlings leaves turned yellow and wilted, and the EL, MDA and H_2_O_2_ levels were increased both in leaves of TRV-0 and TRV-10, whereas these physiological indicators were significantly higher in TRV-10 seedlings than in TRV-0 seedlings (Fig. 6A, B). Exogenous JA treatment could alleviate the damage of drought treatment to seedlings and repress the accumulations of EL, MDA and H_2_O_2_ in oriental melon seedlings under drought conditions (Fig. 6B). These results indicate that exogenous JA could improve plant drought resistance. To explore whether the larger stomatal aperture in TRV-10 seedlings is caused by JA, oriental melon seedlings were treated with 10 μM JA. The stomatal aperture of both TRV-10 plants and TRV-0 leaves became smaller after JA treatment, but there was a lower relative stomatal closure in TRV-10 leaves comparing to that of TRV-0 leaves (Fig. 6C), suggesting that the part of JA-mediated stomatal closure requires the participation of *CmLOX10*. Exogenous JA treatment induced the expression of marker genes (*SLAC1* and *SLAH3*) in regulating stomatal closure in TRV-0 and TRV-10 seedlings, thereby reducing stomatal aperture (Fig. 6D).

### *CmLOX10* expression was feedback regulated by JA signaling

**Figure 7:**
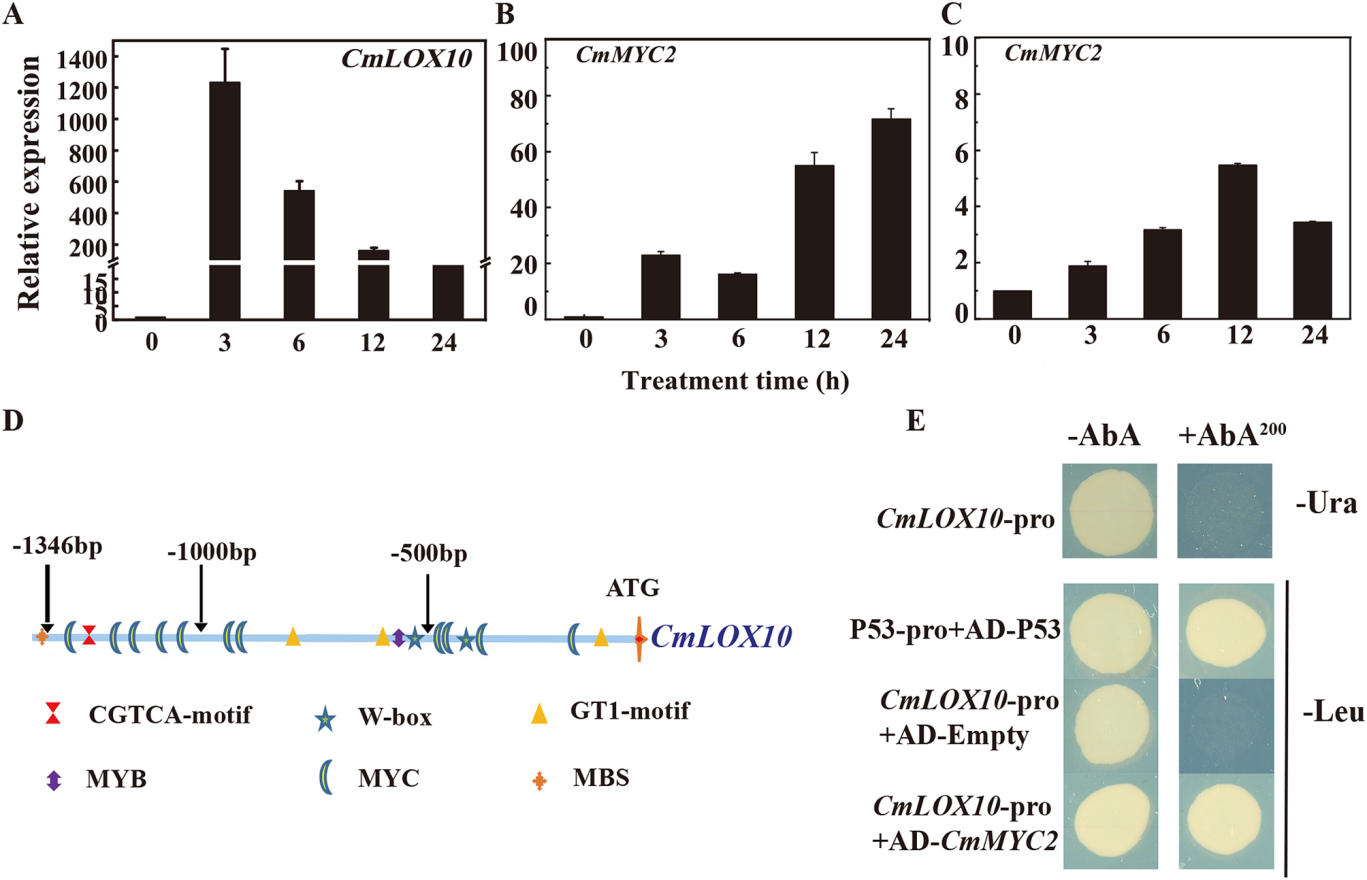
*CmLOX10* is regulated by a core element of JA signaling CmMYC2 in oriental melon. (**A**) Expression of the *CmLOX10* genes under 50 μM JA treatment. (**B**) Expression of the *CmMYC2* genes under 50 μM JA treatment. **(C**) Expression of the *CmMYC2* genes under 8% PEG6000 treatment. (**D**) The distribution of major cis-acting elements existed in the *CmLOX10* promoter. (**E**) YIH was used to analyze the CmMYC2 binding *CmLOX10* promoter. Error bars indicate SD for three measurements. AbA: Aureobasidin A, AbA^200^: the AbA concentration is 200 ng mL^−1^.

Considering that JA biosynthetic pathway was mainly regulated by the transcriptional regulation, especially the regulation of *CmLOX10*, we isolated 1346 bp promoter of *CmLOX10* from ‘YMR’ and analyzed the putative *cis*-regulatory elements using the PlantCARE and PLACE databases. Interestingly, within the *CmLOX10* promoter region, we found a MeJA-responsive element (CGTCA motif), and qRT-PCR analysis showed that *CmLOX10* was induced by JA treatment (Fig. 7A). These results indicate that JA signal transduction pathway might play an important role in *CmLOX10* transcriptional regulation. Therefore, we selected *CmMYC2* (56.09% homology to *AtMYC2*), a core transcription factor in the JA signal transduction pathway, as a candidate gene to investigate the regulation of *CmLOX10* transcription. In this study, *CmMYC2* was induced by JA (Fig. 7B) and PEG6000 (Fig. 7C). In addition, many cis-elements bindings to MYC transcription factor were identified in the 1346 bp promoter region of *CmLOX10* (Fig. 7D). Therefore, we focused on analyzing the potential regulation of *CmLOX10* transcription by *CmMYC2*. The specific binding of *CmMYC2* to *CmLOX10* promoter was verified by Y1H. A 1346 bp promoter sequence upstream of ATG was cloned and constructed into the pAbAi vector. The AbA concentration screening results showed that yeast strain growth was inhibited when SD/-Ura medium supplemented with 200 ng ml^−1^ AbA (Fig. 7E). Subsequently, P53-pro + AD-P53 and *CmLOX10-*pro + AD-*CmMYC2* were transformed into yeast strain and plated on SD/-Leu medium, finding that the yeast strains were still able to grow; however, yeast transformed *CmLOX10-*pro + AD-Empty did not grow on the selection medium (Fig. 7E). These findings demonstrated that the transcription of *CmLOX10* may be directly regulated by *CmMYC2*.

## Discussion

In plants, LOXs have positive roles in abiotic stress responses, especially osmotic stress, salt stress, mechanical damage and drought stress ^22,35^ through a variety of mechanisms. However, the function of LOXs remains largely unknown in oriental melon. In this paper, we found that *CmLOX10* gene, a 13-LOX gene induced by abiotic and hormonal stresses in oriental melon ^30^, was quickly and strongly induced by drought stress (Fig. 1). *CmLOX10*-silenced oriental melon had higher levels of EL, MDA and H_2_O_2_ levels were higher than those of control seedlings after 10 days drought treatment (Fig. 2), which was consistent with the drought-sensitive phenotype of *CmLOX10*-silenced seedlings. In contrast, *CmLOX10*-OX plants had lower content of EL, MDA and H_2_O_2_, supporting the higher survival rates of *CmLOX10*-OX plants than that of WT plants (Fig. 3). Plants respond to drought stress from the cellular to the whole-plant level. Water loss is crucial for plant tolerance to drought stress ^36^. The water loss rate was significantly higher or lower in TRV-10 and *CmLOX10*-OX plants than control, respectively, indicating that the *CmLOX10* plays a positive role in response to drought stress.

RNA-seq analysis was used to examine the possible functions of the *CmLOX10* at the transcription regulation level. Pathway analysis showed that only the hormone metabolism pathway was consistently greater by at least fourfold normalized frequency values in all four comparisons (Table 1). Previous studies indicated that ABA enhanced plant drought tolerance through regulating stomatal movements ^5^. In this study, ABA content and transcript of ABA synthetic genes had no obvious differences between *CmLOX10*-OX and WT plants (Fig. 5C, D). These findings suggest that *CmLOX10* enhanced drought stress resistance, not through the ABA synthesis pathway. Similar results were also found in pepper, in which the expressions of *NCED3* and *NCED5,* ABA biosynthesis genes, showed no significant difference between *CaLOX1*-OX and WT plants under 50 μM ABA treatment ^22^. GO assay showed that “jasmonic acid biosynthetic process” was enriched in the *CmLOX10*-OX line (Fig. 5B). In rice, enhanced α-LeA metabolism improved the ability of drought resistance in drought-tolerant landraces/genotypes ^37^. JA, the metabolite of α-LeA, is a critical signaling molecule in plant defense against biotic and abiotic stresses ^38^. JA accumulation level indicated that *Arabidopsis LOX2*, rice *OsHI-LOX* and *OsLOX1*, and tobacco *NaLOX3* could contribute to JA biosynthesis under wounds and/or herbivores stresses ^39,40^. The connection between JA and drought tolerance has been widely reported in many higher plants ^41^. For example, the content of endogenous JA in plants generally increased under water stress ^42,43^, and exogenous application of JA (or MeJA) at certain concentrations improved drought tolerance ^12,44^. When plants perceive heat stress, activation of *AtOPR3* expression leads to increased JA biosynthesis and accumulation. Subsequently, JA-mediated signaling pathway activated a cascade, resulting in the increase of *DREB2A* expression and enhancing heat tolerance in plants ^45^. Normal wound-induced expression of the JA production genes *ZmLOX8* and *ZmOPR7/8* require LOX10-mediated signaling, whereas normal levels of JA biosynthesis are responses to mechanical damage ^46^. In this study, we discovered that the key genes of JA biosynthesis (*AOC3* and *OPR3*, Fig. 5C, Supplementary Table S2) were upregulated in *CmLOX10*-OX lines under drought stress. Meanwhile, increased JA contents in *CmLOX10*-OX lines were observed compared to WT *Arabidopsis*, decreased JA content in TRV-10 seedlings were also found, compared to TRV-0 (Fig. 5E). These results suggest that *CmLOX10* could play a putative role in drought stress responses by regulating JA synthesis.

JA could be involved in decreasing transpiration loss by regulating stomatal closure ^10,47^. MeJA-induced stomatal closure is an important physiological response process in plants, which has been reported in various plants such as *Arabidopsis* ^48^, barley ^49^, comfrey^50^ and pink blue tobacco ^51^. JA treatment could boost stomatal closure in *Atlox2-1* by inducing higher rates of water loss, being consistent with *AtLOX2* RNAi plants, compared with the WT, and further investigation indicated that the stomata aperture of *Atlox2-1* was wider than that of WT plants under normal growth condition ^52^. In this study, water loss assays on detached leaves showed that there were higher rates of water loss in TRV-10 seedlings with larger stomata aperture, comparing to TRV-0 seedlings (Fig. 4A, C and D). Comparing to WT plants, *CmLOX10*-OX plants had lower water loss rates and smaller stomata aperture (Fig. 4B, C and E). *CaLOX1*-OX plants displayed smaller stomatal aperture than the WT plants under normal and ABA treatment conditions. However, the normalized stomatal aperture of *CaLOX1*-OX plants was not significantly different from the average stomatal aperture of untreated plants, indicating that the low transpiration rate of *CaLOX1*-OX plants was not caused by enhanced ABA-induced stomatal closure, which implied that *CaLOX1* or *CaLOX1*-derived oxylipins might play a role in ABA-independent stomatal closure ^22^. *AtLOX1* and *AtLOX6* were expressed in guard cells, and *AtLOX1* was involved in the regulation of ABA-independent oxylipin-induced stomatal closure at the level of the anion channel *SLAC1* to improve defense immunity ^53^. Our study showed that TRV-10 seedlings accumulated less JA under drought stress with larger stomatal aperture (Fig. 4D, 5E), and displayed smaller stomatal aperture and higher expression of *SLAC1* and *SLAH3* genes in TRV-0 and TRV-10 plants under adding 10 μM JA treatment (Fig. 6D), while *CmLOX10* did not change the sensitivity of *Arabidopsis* to ABA (Supplementary Fig. S2). All these results demonstrate that *CmLOX10* positively regulated drought tolerance through jasmonic acid –dependent pathway in oriental melon.

Interestingly, some studies have shown that JA biosynthesis is also regulated feedback by JA signaling. JA biosynthetic genes *LOX2* and AOS were induced by MeJA in *Arabidopsis* ^24^. Wounding-induced JA levels were significantly decreased in *Arabidopsis coi1-1* mutants ^54,55^ or *COI1*-silenced *Nicotiana attenuate*^56^. Our research found that *CmLOX10* was strongly induced by MeJA (Fig. 7A). Several evidences have pointed out that *Arabidopsis thaliana MYC2* functions as the central role in regulating wounding induced JA accumulation by directly binding to the specific motifs (G-box and its variants) in the promoters of genes involved in JA biosynthesis and catabolism ^25^. The expression of *TomLoxD*, *OPR3*, *AOC*, and *AOS* decreased in the *MYC2*-silenced tomato plants^26^. ChIP-qPCR results indicated that *MYC2* directly bond to the promoters of *LOX2/3/4*, and MeJA treatment enhanced the expression of these genes ^27,28,57^. In this study, we found that, the expression of *CmMYC2* in the oriental melon was strongly induced by MeJA and PEG6000 treatments (Fig. 7B, C). Moreover, many MYC-specific binding sites were found in the promotor of *CmLOX10* (Fig. 7D), and Y1H results showed that CmMYC2 directly targeted *CmLOX10* promoter (Fig. 7E).

In conclusion, this study provided some evidences that *CmLOX10* played an essential role in response to drought stress. *CmLOX10* enhanced drought tolerance by promoting JA accumulation and stomatal closure. Although our results suggested that CmMYC2 could directly bind to *CmLOX10* promoter, and was involved in regulating drought-induced JA biosynthesis; nevertheless, it was absent of direct evidences for the function of CmMYC2, which needs further to be verified.

## Materials and methods

### Plant materials and constructs

*Arabidopsis thaliana* ecotype “Columbia” (WT) were grown in a culture chamber (MLR-350H, SANYO, Japan) at 24 °C, under a 16 h light/8 h dark cycle. The oriental melon *(Cucumis melo* var. *makuwa* Makino) inbred “YMR” using for VIGS were raised in culture chamber (MLR-350H, SANYO, Japan) at 25 °C/18 °C, under a 16 h light/8 h dark cycle.

To specifically silence *CmLOX10*, a 300 bp fragment of the *CmLOX10* conserved coding region was inserted into pTRV2 (TRV-0) to generate pTRV2-*CmLOX10* (TRV-10) according to Liao’s described ^58^. *CmLOX10* gene contains a 2709 bp open reading frame encoding 902 amino acid ^15^ and was inserted into the vector 35S:pB7FWG2. *CmLOX10-*OX plants were generated by using Xing's method ^59^.

### Drought stress treatment

To determine the expression patterns of CmLOXs under drought condition, “Four-leaf stage” oriental melon seedlings treated with 8% PEG6000 were sampled at different times and frozen immediately in liquid nitrogen for RNA preparation.

For drought stress treatment of *Arabidopsis* plants refers to previous study ^59^. For drought stress treatment of oriental melon seedlings, “four-leaf stage” TRV-10 and TRV-0 seedlings were treated by withholding water for 10 days, and the physiological data were measured. For exogenous JA treatment, JA (50 μM) was used for three days before drought treatment.

### Measurements of electrolyte leakage (EL), malondialdehyde (MDA) and ROS content

EL was measured with minor modification s based on McKersie's method. In brief, fresh rosette leaves (0.1 g) of WT and transgenic *Arabidopsis* plants and 10 oriental melon fresh leaves disc (d = 1 cm) were incubated in 20 mL ddH_2_O for one hour at room temperature, determined the conductivities (C1) using a conductivity meter (Model DDS-11A, Rex Instruments Factory, Shanghai, China), subsequently, the leaves were boiled for 10 min, and the conductivities (C2) were recorded after cooling to room temperature. Electrolyte leakage was expressed as C1/C2 × 100%.

In the presence of peroxidase, 3, 3′-diaminobenzidine (DAB) is oxidized by H_2_O_2_ to produce a reddish-brown precipitate. The leaves of *Arabidopsis* and oriental melon treated with drought stress were stained with DAB following the method of Kumar (2014) ^60^.

MDA and H_2_O_2_ contents were determined using MDA Assay Kit (A003-3, Nanjing Jiancheng Bioengineering Institute, China) and H_2_O_2_ Assay Kit (A064-1, Nanjing Jiancheng Bioengineering Institute, China), respectively, following the manufacturer’s instructions.

### Measurements of seed germination and root length

For germination rate and cotyledons greening after different concentrations of ABA (0, 0.5 and 0.75 μM) treatment were scored on the 7rd day after germination, with three replicated assays according to Xing's description ^59^.

For *Arabidopsis* root length experiment, four-day seedlings grown on 1/2 MS medium were transferred to new medium with different concentrations of mannitol (0–500 mM) and ABA (0 and 10 μM) and measured root length after vertical cultured for 7d. For oriental melon root length experiment, “four-leaf stage” plants without watering for 10 days, the roots were observed using root scanner (MICROTEK ScanMaker i800 plus).

### Water loss and stomatal aperture assay

For the measurements of water loss, rosette leaves of 3-week-old *Arabidopsis* (10 plants/ one repeat) and oriental melon (4 plants/one repeat) were detached and weighed immediately (W1), then the plants were left in a plastic tray at 25 °C, 60% relative humidity. The weight (W2) of the leaves was measured at one-hour intervals. The water loss rate was calculated with the following formula: water loss rate (%) = (W1-W2)/W1 × 100%. The experiment repeated three times.

Stomatal aperture measurements were implemented as described previously ^61^. Epidermal peels of expanded mature leaves were floated in stomatal opening solution exposing to light for 2 h, then transferred the epidermal peels into solutions added different concentrations (0, 15%) of PEG6000, JA (0, 10 μM) and ABA (0, 10 μM) for 2 h before being observed. The average of 90 stomata ratio (width/ length) at least repressed stomatal aperture.

### JA and ABA measurement

JA and ABA contents were analyzed using HPLC–MS/MS method according to Xing's description ^59^.

### Transcriptomic analysis

*CmLOX10*-overexpression line OX-17 and WT plants here were used for transcriptomic analyses. Rosette leaves of WT and OX-17 plants were collected from normal and 8d—drought- stress plants for RNA isolation, and each sample was represented by three replicates. RNA quality and quantified were checked using the NanoDrop and 1% agarose gel electrophoresis. A total of 5 μg of RNA was used to synthesize the subsequent sequencing libraries. After library concentration was tested by Qubit 2.0, the library preparations were then sequenced on an Illumina Hiseq platform. After filtering the raw data, Trimmomatic software was used to remove adapter and low-quality reads to get the clean data. Q20, Q30, and the GC contents of the cleaning data were simultaneously calculated. Then we identified the transcript sequence in the reference *Arabidopsis* genome to obtain comprehensive transcript information. RPKM (Reads per Kilobase per Million Reads) was used as a standard of gene expression. And DESeqR package (1.18.0) was used for differential expression analysis of drought stress versus control conditions. To control the false discovery rate, Benjamini and Hochberg’s approach were used to adjust the resulting P-values. The functional analysis of differentially expressed genes (absolute value of log FC > 1 and P-Value < 0.05) were used GO and MapMan as the classification source.

### Quantitative reverse transcription–PCR (qRT-PCR) analysis

Total RNA was isolated using an ultrapure RNA extraction kit (CWbio. Co. Ltd., Beijing, China) and cDNA were synthesized using PrimeScript RT Master Mix (Perfect Real Time) (Takara, Dalian, China) according to the manufacturer's instructions, respectively. Diluted cDNA of fourfold was using for qRT-PCR analysis according to the SuperReal PreMix Plus (SYBR Green) (Takara, Dalian, China) manufacturer’s protocol. The expression analysis was repeated three times. Four oriental melon plants and ten *Arabidopsis* plants, respectively, were used at one biological repeat in statistical analysis. The expression of *Actin7* and *18sRNA* were used as internal reference genes in *Arabidopsis* and oriental melon, respectively. Primers for the qRT-PCR analysis were listed in Table S1.

### Cloned and analysis of *CmLOX10* promoter (*CmLOX10*-pro)

Genomic DNA was extracted from the tender leaves of oriental melon to be used for the cloning of *CmLOX10* promoter searched from the melon genome database (https://melonomics.net/). According to the cloned and sequenced fragments, the putative cis-regulatory elements of *CmLOX10* promoter were performed using PlantCARE (http:// bioinformatics.psb.ugent.be/webtools/plantcare/html/) and PLACE (http:// www.dna.affrc.go.jp/PLACE/) database.

### Yeast one-hybrid (Y1H) assay

Y1H was performed to confirm that the *CmMYC2* can directly regulated *CmLOX10* according to the instructions of Matchmaker Gold Yeast One-Hybrid Library Screening System (Clontech, USA). The *CmLOX10* promoter was subcloned into the pAbAi vector, and the full-length CDSs of *CmMYC2* (MELO3C013851) searched from the melon genome database (https://melonomics.net/) was cloned and inserted into the pGADT7 (AD) vectors. The fusion constructs were transformed into Y1HGold strain, then was cultured on SD/-Ura medium supplemented with (0, 100, 150, 200 ng/ml) Aureobasidin A (AbA), to screen for the concentration of AbA inhibition. Finally, the positive control (P53-pro + AD-P53), negative control (*CmLOX10-*pro + AD-Empty) and the target group (*CmLOX10-*pro + AD-*CmMYC2*) were transformed into yeast strain Y1H to and cultured on SD/-Leu medium containing 200 ng/ml AbA.

### Statistical analysis

All the experiments were carried out three times in a completely random sampling. We applied one-way ANOVAs with SPSS16.0 statistics program, and presented values as means ± SD. Asterisks above the columns indicate significant differences between the TRV-0 and TRV-10 seedlings (Student’s t test, *P < 0.05). All charts were drawing by Origin 8.0 in this paper.

## Supplementary information

Supplementary file1
