# Supplementary material for: CmLOX10 positively regulates drought tolerance through jasmonic acid -mediated stomatal closure in oriental melon (Cucumis melo var. makuwa Makino)
Source: Sci Rep. 2020 Oct 15;10:17452. doi: 10.1038/s41598-020-74550-7 (PMC7562952; doi:10.1038/s41598-020-74550-7)
Supplement: Supplementary file 1 — Supplementary file1 [file 41598_2020_74550_MOESM1_ESM.pdf]

Supplementary material

***CmLOX10* positively regulates drought tolerance through jasmonic acid -mediated stomatal closure in oriental melon (*Cucumis melo* var. *makuwa* Makino)**

Authors: Qiaojuan Xing[1], Jingjing Liao[1], Songxiao Cao[1], Meng Li[1], Tinghui Lv[1], and Hongyan Qi[1]*

Supplementary Table S1. Primer sequences used for qRT-PCR.

Primers	Sequence (5'-3')
**Primers used in *Arabidopsis***	
***AtAOS*-F**	TGGCTTCTATTTCAACCCCTTTTC
***AtAOS*-R**	AGATCCGGTTTCGAGATGACTTG
***AtAOC3*-F**	AACTGAGCGTGTACGAAATCAAT
***AtAOC3*-R**	CAAACATACTGCATTCACAAGGA
***OPR3*-F**	TGGACGCAACTGATTCTGAC
***OPR3*-R**	GCGAGCTTTGAGCCATTAAC
***Actin7*-F**	GGAACTGGAATGGTGAAGGCTG
***Actin*7-R**	CGATTGGATACTTCAGAGTGAGGA
***CmLOX10*-F**	GTCACGGCGGTTTTGAACATA
***CmLOX1*0-R**	GATTTCGGTCAGCATTCCTTTC
**Primers used in oriental melon**	
***18S-RNA*-F**	GTGATGGTGTGAGTCACACTGTTC
***18S-RNA*-R**	ACGACCAGCAAGGTCCAAAC
***CmLOX01*-F**	CCATCAACTTATCAGCCATT
***CmLOX01*-R**	GTTCGTTCAAGAAGACCAT
***CmLOX02*-F**	TAGCACCGAAGGAATCAC
***CmLOX02*-R**	AGACAGCACAATAACAGAGT
***CmLOX03*-F**	GACGACGAGAATGGAGAG
***CmLOX03*-R**	GCTGGTTGAACTGTTGATAC
***CmLOX04*-F**	GGCTAACTTCAATCCCACCA
***CmLOX04*-R**	GCTCAGTGAAGTTATCAAGA
***CmLOX05*-F**	GCTGCTTGTTCCTCATTA
***CmLOX05*-R**	AGTCTTCAACTGCCATTC
***CmLOX06*-F**	GTGTATGTTCCAAGAGATG
***CmLOX06*-R**	TGAATAAGTTGAGGAGTA
***CmLOX07*-F**	TACTTGGAGGAATGGATA
***CmLOX07*-R**	TTGTTGTAACGGTGAGAC
***CmLOX08*-F**	GGTAACTGGTCGTGGAAT
***CmLOX08*-R**	AAGGCAGAGGAATAACAGAA
***CmLOX09*-F**	CAGATCCATCTTGTGAAC
***CmLOX09*-R**	AGTTGGTAGAGTCATTCC
***CmLOX10*-F**	TGACAGGACAAGGAGTTC
***CmLOX10*-R**	CGGTATTGGCAAGAATGTTA
***CmLOX11*-F**	CAAGTCATTCTCCAGATG
***CmLOX11*-R**	GTTGATAAGGTCCAATCC
***CmLOX12*-F**	GTTAAGTTCTTCAGCATACG
***CmLOX12*-R**	ACGAGGATGGATAGTAATG
***CmLOX13*-F**	CAAGCAACACAGGTAATG
***CmLOX13*-R**	CTCCAGTTCTATTCTTCAAG

***CmLOX14*-F**	CAAGTGAACCAGATAACAAG
***CmLOX14*-R**	CAGAGGAATTGGAATGAAG
***CmLOX15*-F**	CTATTATGCTGATGCTGAG
***CmLOX15*-R**	GAAGGAAGTTGACAGATG
***CmLOX16*-F**	ATACGGACCTCAAGAATC
***CmLOX16*-R**	GAGTCAAAGTGTCATCAG
***CmLOX17*-F**	TGACTATCTAATGCCACTTC
***CmLOX17*-R**	CCAACTTATCTCTTCTCCT
***CmLOX18*-F**	TGGAGACTATCACAATCG
***CmLOX18*-R**	CTTTCCCATCACCTCTAA
***SLAC1*-F**	TTTCCCCGTTCATCAACCT
***SLAC1*-R**	GCCTCCAACCACCCACAT
***SLAH3*-F**	CCCTGGATAGCCCTCTTGTT
***SLAH3*-R**	TCTCATTGGTTGGTAGTCTCTGAT

Supplementary Table. S2 Expression of plant hormone JA and ABA biosynthesis gene transcripts (RNA-seq analyses).

	Gene Symbol	CK-10			CK-WT			D-10			D-WT			Log_2FC			
														CK-10 /CK-WT	D-10/ D-WT	D-10/ CK-10	D-WT /CK-WT
JA biosynthesis	*CYP74A*	227.14	831.13	224.96	609.06	448.47	388.96	118.34	266.35	251.53	153.20	247.71	205.76	-0.17	0.07	-1.01	-1.25
	AOC1	116.17	438.18	169.34	211.62	115.23	141.75	57.03	209.37	124.33	122.61	176.14	120.90	0.63	-0.10	-0.89	-0.16
	AOC2	113.09	417.27	176.53	216.77	154.06	174.19	69.82	191.83	114.61	102.77	184.51	136.32	0.38	-0.17	-0.91	-0.36
	AOC3	27.92	96.31	16.19	24.63	50.42	68.67	7.90	9.74	23.48	7.51	5.35	6.55	-0.03	1.08	-1.77	-2.89
	AOC4	42.21	30.69	19.47	46.94	53.43	42.94	49.96	26.33	26.59	34.73	24.31	34.92	-0.63	0.13	0.16	-0.61
	OPR3	141.14	678.90	92.14	217.14	168.87	238.16	53.24	74.77	142.77	36.65	34.92	44.65	0.55	1.22	-1.75	-2.43
ABA biosynthesis	*ZEP*	468.63	580.51	641.12	524.18	456.60	497.33	397.22	557.86	478.78	536.44	809.83	520.77	0.19	-0.38	-0.24	0.34
	ABA2	24.02	14.12	19.41	28.08	23.50	20.26	22.68	12.75	12.19	18.74	15.34	19.23	-0.32	-0.16	-0.27	-0.43
	ABA4	14.15	13.70	19.92	25.50	18.65	17.17	16.39	16.90	14.92	16.97	25.80	25.32	-0.36	-0.50	0.01	0.15
	AAO3	6.75	8.38	7.68	5.13	5.90	8.56	8.58	8.46	11.31	13.88	8.68	6.08	0.22	-0.02	0.31	0.55
	NCED3	4.09	5.52	6.75	7.74	10.59	16.95	8.40	8.45	15.17	17.38	12.40	6.91	-1.11	-0.20	0.97	0.06
	NCED5	0.06	0.11	0.20	0.08	0.12	0.09	0.08	0.00	0.11	0.19	0.04	0.04	0.37	-0.49	-0.97	-0.10

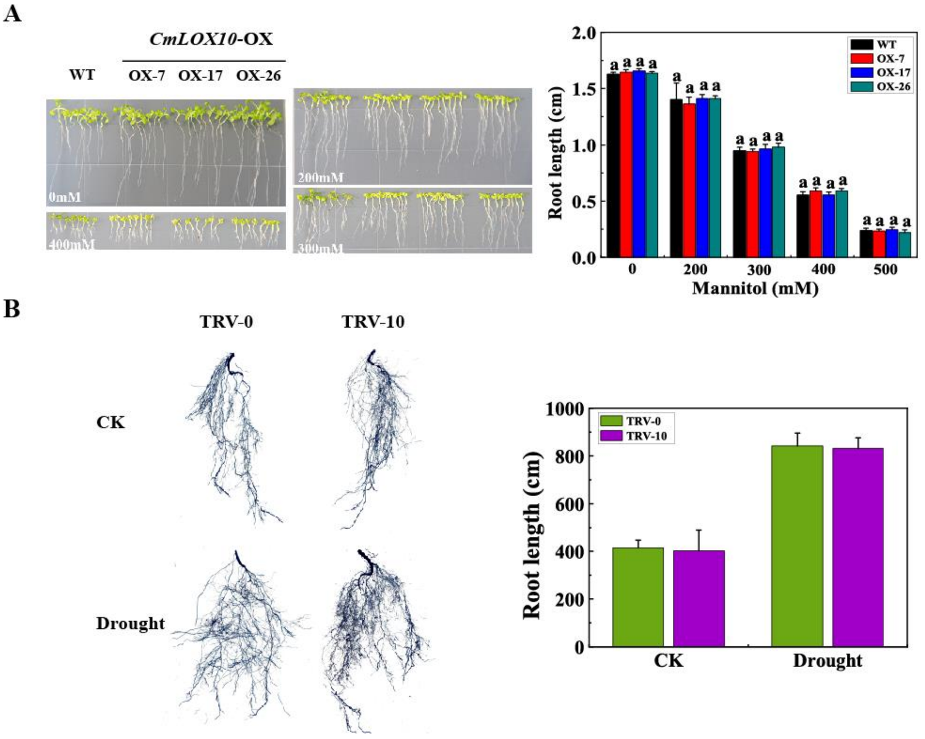

Supplemental Fig. S1 *CmLOX10* has no effect on root length. (**A)** Phenotype and root length of *CmLOX10*-overexpressed plants in the presence of different concentrations of mannitol. (**B)** Phenotype and root length of *CmLOX10*-silenced and control plants under normal and drought conditions. Error bars indicate SD for three measurements. Different letters indicate significant differences at P<0.05 (Duncan's multiple range test) of levels for the same index in WT, OX-7, OX-17 and OX-26 plants.

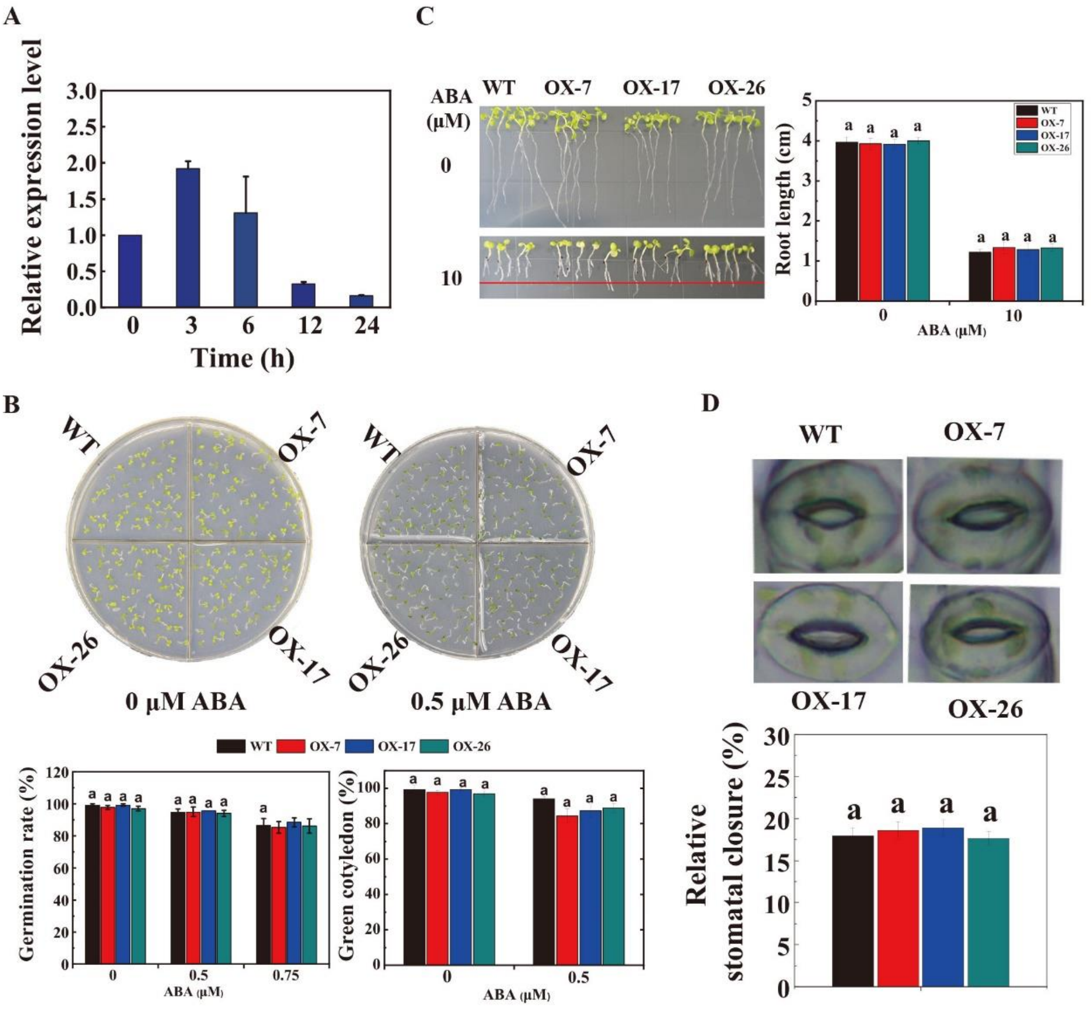

Supplemental Fig. S2 *CmLOX10* does not respond to ABA signals. (**A)** The "four leaves stage" oriental melon leaves were treated with 100 μM ABA then expression of the *CmLOX10* gene was measured. The experiment including three biological repeats, and four plants were used at one biological repeat (**B)** Phenotype, germination rate and green cotyledon and (**C)** Root length of WT and *CmLOX10*-OX plants under ABA treatment. (**D)** Relative stomatal closure indicates the percentage of reduction of stomatal aperture after ABA (10 μM) treatment compared with non-treated leaf. Error bars indicate SD for three measurements. Different letters indicate significant differences at P<0.05 (Duncan's multiple range test) of levels for the same index in WT, OX-7, OX-17 and OX-26 plants.
